# Research on Novel CMUTs for Detecting Micro-Pressure with Ultra-High Sensitivity and Linearity

**DOI:** 10.3390/mi12111340

**Published:** 2021-10-30

**Authors:** Qi Ding, Hongliang Wang, Hanqiang Zhang, Xiao Huang, Xiaolei Sun, Zhenjie Qin, Rui Ren, Jiajun Zhu, Changde He, Wendong Zhang

**Affiliations:** National Key Laboratory for Electronic Measurement Technology, Key Laboratory of Instrumentation Science & Dynamic Measurement, Ministry of Education, North University of China, Taiyuan 030051, China; s1906184@st.nuc.edu.cn (Q.D.); s1906104@st.nuc.edu.cn (H.Z.); huangxiao.nuc.edu@outlook.com (X.H.); s1906047@st.nuc.edu.cn (X.S.); s2006116@st.nuc.edu.cn (Z.Q.); s2006084@st.nuc.edu.cn (R.R.); s2006195@st.nuc.edu.cn (J.Z.); hechangde@nuc.edu.cn (C.H.); wdzhang@nuc.edu.cn (W.Z.)

**Keywords:** capacitive micromachined ultrasonic transducer (CMUT), frustum cone-like cavity, slotted membrane, pressure sensitivity

## Abstract

Capacitive micromachined ultrasonic transducers (CMUTs) have been indispensable owing to their resonance characteristics in the MHz frequency range. However, the inferior pressure sensitivity and linearity of traditional CMUTs themselves cannot meet the actual demands of micro-pressure measurements. In this paper, two novel CMUTs are proposed for the first time to improve the measuring performance of micro-pressure in the range of 0–10 kPa. The core concept of the enhancement is strengthening membrane deformability by partly adjusting the CMUT framework under the combined action of electrostatic force and uniform pressure. Two modified structures of an inverted frustum cone-like cavity and slotted membrane are presented, respectively, and a finite element model (FEM) of CMUT was constructed and analyzed using COMSOL Multiphysics 5.5. The results demonstrate that the maximum displacement and pressure sensitivity are improved by 16.01% and 30.79% for the frustum cone-like cavity and 104.22% and 1861.31% for the slotted membrane, respectively. Furthermore, the results show that the width uniformity of the grooves does not influence the characteristics of the membrane, which mainly depend on the total width of the grooves, greatly enriching design flexibility. In brief, the proposed structural designs can significantly improve the micro-pressure measurement performance of the CMUT, which will accelerate the rapid breakthrough of technical barriers in the fields of aerospace, industry control, and other sensing domains.

## 1. Introduction

As a fundamental physical parameter, the precise measurement of pressure is crucial in the areas of industrial production, scientific research, national defense science and technology, biomedicine, and other engineering disciplines. In particular, in a micro-pressure environment, it has been one of the most crucial techniques for the design of sensors to measure weak pressure efficiently and accurately. Coupled with pressure mutation, the development of practical sensors capable of detecting pressure with high sensitivity and linearity is currently lacking [1]. However, micro-pressure sensors have remained the focus of research owing to their exigent needs in the fields of aerospace, industry control, and others. For example, applications include the height measurement of aircraft through high-altitude pressure [2], pressure condition monitoring of chemical reaction processes in industrial manufacture [3], pressure detection of external pulsating wind in high-speed rail operation systems [4], and pressure difference measurement of the internal carotid artery before and after surgery [5].

In this case, various pressure sensors have been devised and put into use for a long time, the most prominent of which belongs to pressure sensors based on MEMS (Micro-electromechanical Systems) processes. Compared with other sensors, MEMS pressure sensors possess distinct advantages such as miniaturization, high resonance frequency, high sensitivity, low noise, and mass production [6]. MEMS pressure sensors can be roughly divided into three categories: piezoresistive, capacitive, and resonant [7,8,9]. Piezoresistive pressure sensors, which are prepared using the piezoresistive effect of the material, have a simple manufacturing process and satisfy linearity. However, their inherent sensitivity to temperature dramatically decreases their measurement precision, which reduces their widespread application [10]. The capacitive pressure sensor utilizes the change in capacitance between the upper and lower plates to achieve pressure detection, resulting in low temperature drift, low power consumption, and high sensitivity. Nevertheless, complex post-processing circuits are needed to eliminate parasitic effects that arise from intrinsic nonlinearity and small output capacitance [11]. The resonant pressure sensor performs pressure measurement indirectly through a shift in the resonance frequency. In contrast, the resonant pressure sensor has higher sensitivity, precision, and anti-interference because it is mainly affected by the characteristics of the mechanical structure of the device. Consequently, resonant pressure sensors have emerged as promising candidates for higher-level sensing domains. Among all the sensors, the typical representative device is the silicon micro-resonant MEMS pressure sensor [12].

Owing to its resonant characteristics in the MHz frequency range, the capacitive micromachined ultrasonic transducer (CMUT) is an indispensable component of silicon micro-resonant MEMS pressure sensors. When designed as a pressure sensor, the CMUT can measure static or slowly varying pressures. In contrast to the resonant pressure sensors of the second sensitive element, such as the cantilever beam, the CMUT membrane can directly sense pressure, which effectively improves the resolution of CMUT measurements. The high resonance frequency and quality factor of CMUTs enable measurements with higher sensitivity. In addition, the stable structure, good impedance matching, and batch production of CMUTs ensure the reliability of pressure measurements in complex environments [13].

Based on the facts above, using CMUTs as resonators has been extensively researched in recent decades. Kagawa [14] operated a CMUT in normal mode and demonstrated its functionality. The frequency decreased as the uniform pressure increased. Considering the large deflections of the membrane, Schuster [15] found that when the displacement state reached a large deformation, the frequency increased with increasing pressure. However, both neglected the effect of the electrostatic force on the resonant frequency. Vogl and Nayfeh [16] specifically studied the effect of bias voltage by establishing a reduced-order model, but failed to achieve conclusive results. Later, Talebian et al. [17] employed the Galerkin method to derive a numerical solution that clearly demonstrated the effect of the electrostatic force on the resonant frequency. In 2013, Li et al. [18] derived an approximate solution of the resonant frequency with two loads simultaneously acting on the membrane, using the energy equivalent method. All these results have laid a solid theoretical foundation for the application of CMUTs to pressure detection, which is useful for the present study. Li et al. [19] presented a new approach and applied CMUTs for the measurement of ultra-low pressure (below 1000 Pa). Further research was carried out by the team, and the results verified that the resonance frequency has a linear relationship with the pressure under the action of the bias voltage, and the air damping effect could be negligible. In addition, a temperature compensation method was proposed to eliminate the negative influence of the external ambient temperature [20]. Zhang et al. [21] developed a CMUT array which featured a sensitivity of 50.57 Hz/kPa in the pressure range of 0–10 kPa and 441.08 Hz/kPa in the pressure range of 20–101 kPa. Later, a novel dual-frequency CMUT that can simultaneously detect pressure and humidity was proposed for the first time by the group, which had good experimental performance [22]. The studies above demonstrated the feasibility of using CMUTs for pressure measurements.

However, these studies did not fully meet application sensing requirements. Therefore, the development of efficient pressure sensors is necessary to further improve the measurement accuracy and electromechanical conversion rate of CMUTs. For this, the most common and efficient method is modifying the structure of CMUTs. Zhou et al. [23] significantly promoted the capacity of deformation and work efficiency of a CMUT by introducing slots at the edge of the membrane and changing the supporting structure to a ‘dog leg’ shape. Jiang et al. [24] presented two novel membrane structures, demonstrating that slotted and corrugated membranes can greatly improve the efficiency of CMUT. However, these studies failed to perform research related to pressure. To date, there have been no further reports on the enhancement of pressure sensitivity of CMUT. In this paper, two novel CMUTs are proposed for micro-pressure measurements in the range of 0–10 kPa; they all improved the pressure measurement performance of the CMUT by further optimizing its structural design.

In this paper, Section 2 focuses on the basic theory and unique structure design of CMUTs. In Section 3, we cover the optimization of the geometrical parameters of CMUTs by the FEA. Additionally, the displacement, pressure sensitivity, resonance frequency, and R-square are compared with traditional CMUTs to highlight the advantages of new structures. In addition, the rationality of the calculation results is further supported through comparison with previous studies.

## 2. Theory and Design

### 2.1. Basic Theory of CMUT for Micro-Pressure Detection

**Figure 1:**
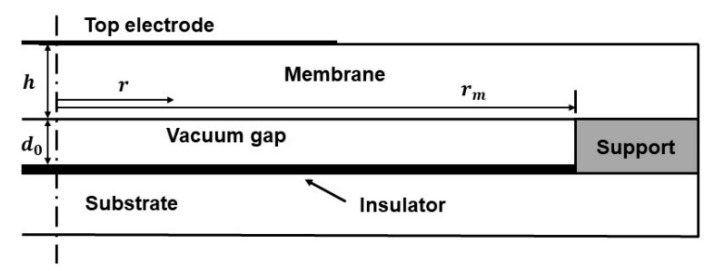
Schematic diagram of a typical capacitive micromachined ultrasonic transducer (CMUT) cell (C1).

Capacitive micromechanical ultrasonic transducer (CMUT) cells for micro-pressure measurement are composed of a metal upper electrode, vibrating membrane, support, vacuum cavity, insulator, and heavily doped substrate (fixed bottom electrode) [25]. The membrane is flat, which can generally be designed as circular, rectangular, or hexagonal [26]. For the sake of minimizing the effect of stress concentration, the typical circular CMUT was adopted as a resonator in this paper, as shown in Figure 1.

The edge of membrane—on top of which a metal electrode of radius $r_{e}$ is deposited—is clamped, and a sealed vacuum exists between the membrane and the substrate. Without any initial deformation and external force, the basic resonance frequency of the CMUT can be expressed as follows [27]:

(1) $$f_{0}=\frac{10.21}{2\pi r_{m}{}^{2}}\sqrt{\frac{D}{\rho h}}$$

The flexural stiffness of the membrane is defined as follows [28]:

(2) $$D=Eh^{3}/\left[12\left(1-v^{2}\right)\right]$$

where $r_{m}$ and h are the radius and thickness of the membrane, respectively; and E, v, and ρ are Young’s modulus, Poisson’s ratio, and the density of the membrane material, respectively. Based Equation (1), $f_{0}$ is mainly affected by the material properties and geometric parameters of the membrane. The frequency is proportional to the thickness h and is inversely proportional to the radius $r_{m}$. Furthermore, the resonance frequency can also be adjusted by changing the external load. In this paper, the external load mainly consists of the DC bias voltage $V_{\mathrm{dc}}$ and pressure P acting on the membrane.

The membrane will deform downward under the combined action of uniform pressure and electrostatic force, and the deformation can be divided into large and small. For a deflection-to-thickness ratio of <0.2, the deflection is treated as a small deflection; otherwise, it is treated as a large deflection. Once the deflection of the membrane is small enough and the ratio of the diameter to the thickness of the membrane is greater than 20, the small deflection assumption and the classical Kirchhoff thin plate theory can be invoked [29]. Then, the resonant frequency of the CMUT can be obtained as follows:

(3) $$f_{P}=f_{0}\left(a-bP\right)$$

where a reflects the effect of the bias voltage on the resonant frequency of the CMUT, namely the spring softening effect, which is a function of the bias voltage, indicating that the resonant frequency of the CMUT shifts as the bias voltage increases. As the top electrode moves closer to the bottom electrode due to the presence of the bias voltage, the electrical field increases and the top electrode displaces further, acting as if the spring constant of the top electrode decreases under the influence of the applied voltage [30]. The parameter b represents the frequency shift caused by pressure change at a given DC bias voltage. Detailed expressions for a and b are given in [14]. The corresponding pressure sensitivity is defined as follows [14]:

(4) $$S_{P}=\frac{1}{af_{0}}\frac{\partial f_{P}}{\partial P}=-\frac{b}{a}\left({\mathrm{ppm}\;\mathrm{Pa}}^{-1}\right)$$

Equation (4) indicates that $S_{P}$ mainly depends on the applied bias voltage. A larger bias voltage will lead to higher pressure sensitivity. Thus, an effective measure to improve the pressure sensitivity of a CMUT is to make the applied bias voltage less than and as close as possible to the collapse voltage. As an important design parameter of CMUTs, the collapse voltage should not be so high that the high DC bias voltage required in micro-pressure measurement cannot be obtained. The collapse voltage is expressed as follows:

(5) $$V_{\mathrm{collapse}}=\sqrt{\frac{8Kd_{0}{}^{3}}{27\varepsilon_{0}A}}$$

where K is the equivalent spring coefficient of the membrane, A is the area of the electrode, $d_{0}$ is the cavity height, and $\varepsilon_{0}$ is the vacuum dielectric constant. Additionally, $V_{\mathrm{collapse}}$ is inversely proportional to the radius of the membrane and is proportional to the cavity height, based on Equation (5). The displacement of the membrane at $V_{\mathrm{collapse}}$ is 1/3 of the cavity height $d_{0}$, which is the maximum deformation permitted by the membrane [31].

### 2.2. Design of the Structure

The characteristics of CMUTs are closely related to the structure, material, and loads, which indirectly affect the pressure measurement performance by changing the displacement of the membrane. The most prominent among them is the resonance structure of a CMUT. As shown in Figure 1, the traditional circular CMUT for micro-pressure detection mainly consists of a flat membrane and cylindrical cavity, which is denoted as C1 here. When electrostatic force and uniform pressure work together, the initial displacement at the radial position r is as follows:

(6) $$w\left(r\right)=\frac{Q+P}{64\left[D-Qr_{m}{}^{4}/\left(42d_{0}\right)\right]}{\left(r_{m}{}^{2}-r^{2}\right)}^{2}$$

where the initial deflection is zero. The electrostatic force per unit area can be written as follows:

(7) $$Q=\varepsilon_{0}V_{\mathrm{dc}}{}^{2}/\left(2d_{0}{}^{2}\right)$$

When the structure, materials and loads of a CMUT change, the displacement of the membrane changes as well [18]. Assuming the measured pressure P to be constant, it can be seen from Equation (6) that the flexural stiffness and electrostatic force have contrasting effects on the displacement of the membrane. This indicates that an increase in electrostatic force will promote the deformation of the membrane, and that an increase in the flexural stiffness will produce a larger membrane displacement. Moreover, the flexural stiffness is closely related to the structural design, and the cavity height influences the electrostatic force. Therefore, optimizing the structural design of a CMUT has become an important means for improving its overall performance. In addition, the traditional CMUT has several deficiencies, such as a low electromechanical conversion rate. This study introduces novel CMUTs with unique structural designs for the cavity and membrane, which can be used for micro-pressure detection.

#### 2.2.1. CMUT of Inverted Frustum Cone-Like Cavity

**Figure 2:**
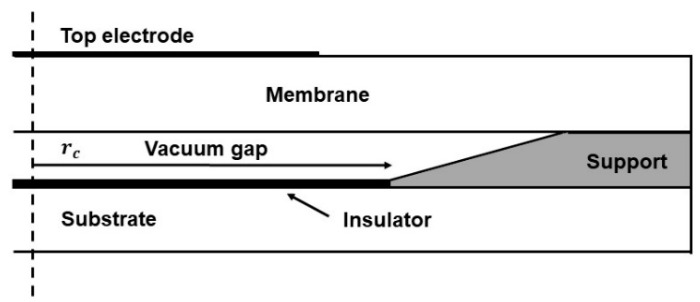
Schematic diagram of C2 (CMUT with an inverted frustum cone-like cavity).

Based on C1, the cavity bottom radius was reduced to $r_{c}$ to form an inverted frustum cone-like cavity, thereby lowering the average height of the sealed cavity. Assuming that the bias voltage is fixed, the electrostatic force is boosted; in response, the displacement of the membrane increases, according to Equation (6). Thus, it can be derived from Equation (5) that the collapse voltage of the CMUT decreases, which is beneficial for micro-pressure detection. In this paper, the CMUT with an inverted frustum cone-like cavity is denoted as C2 in Figure 2.

#### 2.2.2. CMUT of Slotted Membrane

**Figure 3:**
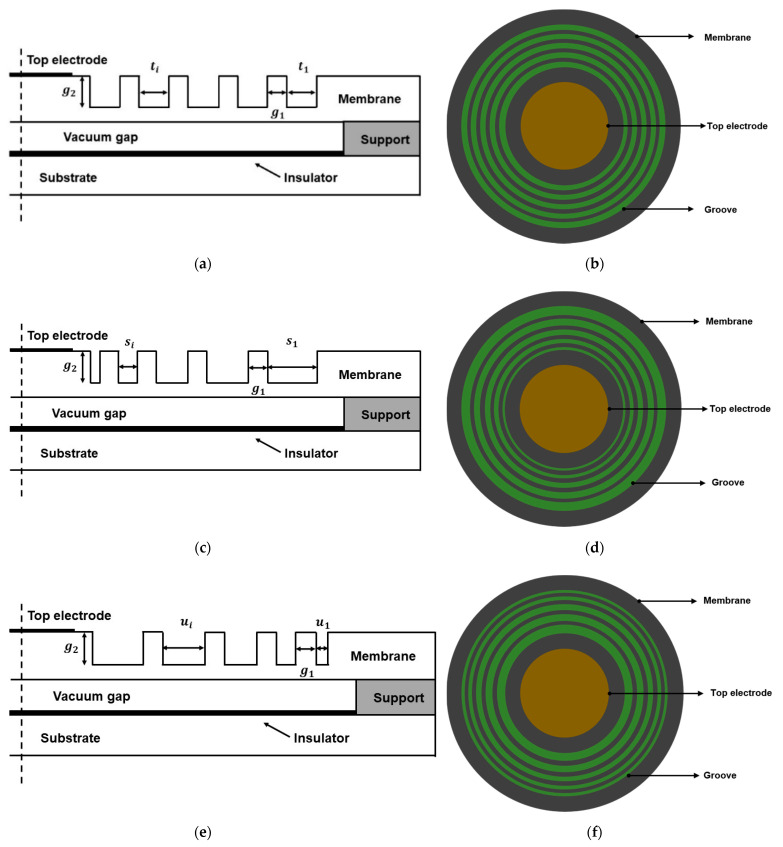
Schematic diagrams of different CMUT designs: C3, C4, and C5: (**a**) front view of C3; (**b**) top view of C3; (**c**) front view of C4; (**d**) top view of C4; (**e**) front view of C5; (**f**) top view of C5.

Based on C1, slots were introduced on the surface of the membrane, thereby forming several annular grooves, which not only reduce the average thickness of the membrane, but also decrease its effective flexural stiffness in accordance with Equation (2). This contributes to enhancing the deformation capacity of the membrane. Due to the diversity of grooves and distribution, three different CMUTs are proposed in this paper: C3, C4, and C5, as shown in Figure 3a–f, respectively. The groove widths of C3 were $t_{i}\;$(i= 0, 1 … n), where $t_{1}\;$=$\;t_{2}\;$= … =$\;t_{n}$; those of C4 were $s_{i}$ (i = 0, 1 … n), where $s_{1}\;$>$\;s_{2}\;$> … >$\;s_{n}$; and those of C5 were $u_{i}$ (i = 0, 1 … n), where $u_{1}\;$<$\;u_{2}\;$< … <$\;u_{n}$. In addition, n,$\;g_{1}$, and $g_{2}$ in C3–C5 are the number, separation distance, and depth of the grooves, respectively; $g_{1}$ and $g_{2}$ do not vary across C3, C4, and C5. In addition, the total widths of the grooves in C3, C4, and C5 are equal; that is, $t_{1}\;$+$\;t_{2}$+ … +$t_{n}\;$=$\;s_{1\;}$+$\;s_{2}$+ … +$\;s_{n}\;$=$\;u_{1}\;$+$\;u_{2}\;$+ … +$\;u_{n}$.

## 3. Simulation and Comparison of CMUTs

### 3.1. Simulation and Analysis

To facilitate the optimization of geometric parameters and characterization of performance for the CMUTs in the process of micro-pressure measurement, a three-dimensional (3D) finite element model (FEM) was constructed in COMSOL Multiphysics 5.5 (COMSOL. Inc., Burlington, MA, USA) to simulate the characteristics of the CMUT cell. All models coupled the electromechanical domain to the vacuum cavity and solid materials. The silicon membrane was fixed around to prevent lateral movement but permit deflection. All mechanical and electrical properties (density ρ, Young’s modulus E, Poisson’s ratio v, and permittivity $\varepsilon_{\gamma }$) of the materials in this paper are listed in Table 1.

Due to the limitation of the material itself and the subsequent processing requests, the radius of the membrane was set to 200 μm. Furthermore, the thickness of the insulation layer was set to 2 μm to ensure good electrical isolation between the top electrode and the conductive silicon substrate. Furthermore, the bias voltage was set to 200 V to guarantee the normal operation of all models, thereby preventing the membrane from collapsing due to collapse voltage discrepancies. Overall, setting these parameters contributed to the diversity of the mechanical structure.

#### 3.1.1. Simulation of C1

**Figure 4:**
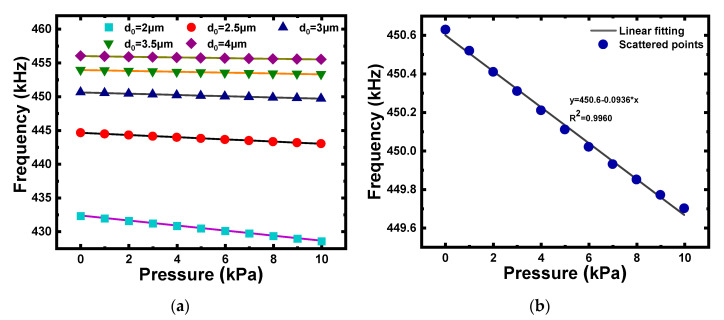
(**a**) Resonance frequency of C1 with different cavity heights of 2 μm, 2.5 μm, 3 μm, 3.5 μm, and 4 μm for pressure detection in the range of 0–10 kPa. (**b**) Resonance frequency of C1 with different cavity heights of 3 μm for pressure detection in the range of 0–10 kPa.

**Figure 5:**
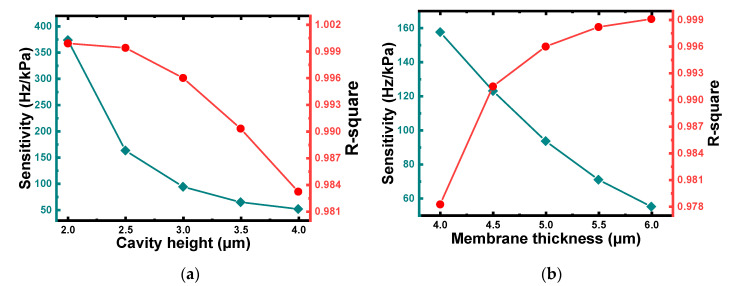
(**a**) Pressure sensitivity and linear fit of C1 with cavity heights of 2 μm, 2.5 μm, 3 μm, 3.5 μm, and 4 μm and membrane thickness of 5 μm under pressures of 0–10 kPa. (**b**) Pressure sensitivity and linear fit of C1 with membrane thicknesses of 4 μm, 4.5 μm, 5 μm, 5.5 μm, and 6 μm and cavity height of 5 μm under pressures of 0–10 kPa.

The thickness of the membrane and the cavity height are important parameters that are strongly associated with the performance of a CMUT. According to the theoretical analysis in Section 2.1, the collapse voltage of the CMUT decreases as the cavity height decreases, and the bias voltage should be as close as possible to the collapse voltage. Thus, a smaller cavity height was preferred for reducing the collapse voltage of the CMUT. Assuming a membrane thickness of 5 μm, C1 with different cavity heights of 2 μm, 2.5 μm, 3 μm, 3.5 μm, and 4 μm under pressures of 0–10 kPa were simulated using the finite element method. As illustrated in Figure 4, the resonance frequency of C1 decreased as the pressure acting on the membrane increased, with a relatively good linear relationship. Furthermore, a larger cavity height led to a higher pressure sensitivity and improved linear fit, as shown in Figure 5a. Considering the larger displacement generated from C2–C5, a 3 μm depth cavity was selected for micro-pressure detection.

Then, assuming a cavity height of 3 μm, C1 with different membrane thicknesses of 4 μm, 4.5 μm, 5 μm, 5.5 μm, and 6 μm under pressures of 0–10 kPa were simulated. As illustrated in Figure 5b, the variation trends of the pressure sensitivity and linear fit with the thickness of the membrane were the complete opposite. As a compromise, a 5 μm-thickness membrane was selected.

**Figure 6:**
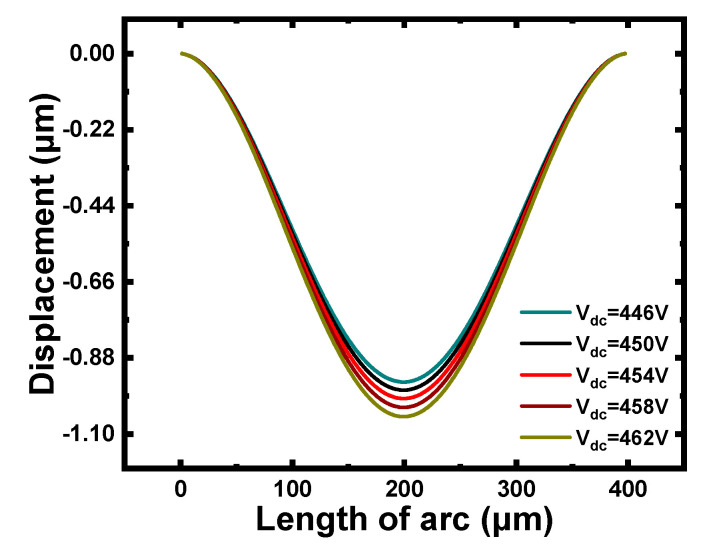
The displacement curves of C1 with different bias voltages.

The initial deflection of the membrane was the largest under a pressure of 10 kPa, where the collapse voltage of the CMUT was the smallest. Considering normal operation of all models, the pressure of 10 kPa was utilized to simulate the collapse voltage of C1. Figure 6 shows that the membrane displacement of C1 was close to 1 μm at 454 V, which was regarded as the critical voltage value of C1.

#### 3.1.2. Simulation of C2

**Figure 7:**
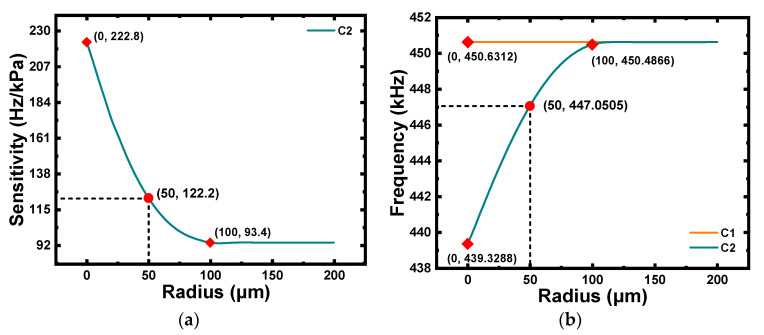
(**a**) Pressure sensitivity of C2 under pressures of 0–10 kPa (0 μm ≤$\;r_{c}\;$ ≤ 200 μm ). (**b**) Comparison of resonant frequencies of C1 and C2 under pressure of 0 Pa (0 μm  ≤$\;r_{c}\;$ ≤ 200 μm ).

To determine the performance characteristics of C2 with cavities of different sizes, the bottom radius $r_{c}$ had to be increased gradually at a spacing of 10 μm in the range of 0–200 μm, and the acquired CMUTs were simulated under pressures of 0–10 kPa. As displayed in Figure 7a, the pressure sensitivity decreased rapidly as the bottom radius increased and was effectively stable at 93.4 Hz/kPa, where the bottom radius of the cavity was just half the radius of the membrane. It can be concluded from Figure 7b that a larger bottom radius would result in a smaller decrease in the resonance frequency of C2 compared to C1 under a pressure of 0 Pa; that is, the resonance frequency of C2 increased with the increase in the bottom radius in the range of 0–100 μm. Thus, the frequency remained effectively unchanged when the bottom radius exceeded 100 μm. Therefore, the radius of the bottom cavity should not be too large or too small. An excessively large radius may lead to a significant pressure sensitivity reduction, while an excessively small radius may result in a sharp reduction of the membrane’s resonance frequency. In summary, C2 with a bottom cavity of 50 μm was chosen for micro-pressure detection.

**Figure 8:**
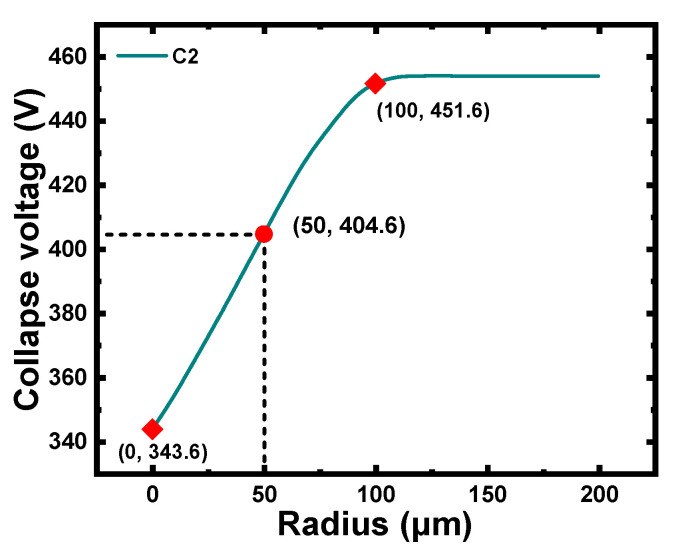
Collapse voltage of C2 with different values of bottom radius of cavity under pressure of 10 kPa (0 μm ≤$\;r_{c}\;$ ≤ 200 μm ).

In this part, the variation in the collapse voltages of C2 along with the bottom radius of the cavity was studied. Figure 8 illustrates that the collapse voltage of C2 increased as the radius increased, which was mainly attributed to the decrease in the effective height of the cavity caused by the reduction in the bottom radius of the cavity. Furthermore, the variation in the collapse voltage was no longer notable beyond 100 μm. The collapse voltage of C2 with a bottom radius of 50 μm was 404 V, which was higher than the bias voltage applied in the micro-pressure detection.

#### 3.1.3. Simulation of C3–C5

Apart from the radius and thickness, the width, depth, number of grooves, and the distance between adjacent grooves are non-negligible factors that dramatically affect the characteristics of slotted membranes. Therefore, a reasonable selection of these parameters was essential for C3–C5. As reported, the working efficiency of the CMUT increased as the radial position of the grooves increased; thus, the lifting efficiency approaching the center of the membrane was gradually weakened. Hence, the position of the annular groove should be set as far as possible from the edge of the membrane [22]. Considering the stability of the membrane after introducing slots, r = 195 μm was set as the initial position of the grooves at the margin of the membrane.

Because the radial position was independent of the groove depth, the one-groove CMUT was first analyzed for an optimum groove depth. Assuming C3 with a single groove with a width of 2 μm, a groove depth of 0.5–4.5 μm was applied for pressure detection in the range of 0–10 kPa. According to the data in Table 2, the pressure sensitivity and linear fit of C3 continuously increased as the depth of the grooves increased, and the linear relationship remained around an ideal state when surpassing 3.5 μm. Therefore, a larger groove depth was preferred to maximize the pressure sensitivity. However, an excessive groove depth yielded a larger membrane displacement, which would greatly accelerate the failure of the small deflection theory and the collapse of the membrane. These aspects, in addition to the reliability of the mechanical structure and limitation of the number and width of grooves, resulted in the selection of a groove with a depth of 3.5 μm in this study.

**Figure 9:**
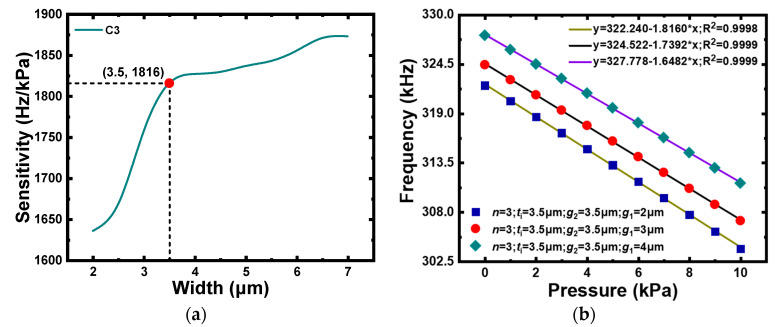
(**a**) Pressure sensitivity of C3 with different groove widths (*n* = 3, $g_{1}\;$= 3.5 μm, $g_{2}\;$ = 3.5 μm ). (**b**) Resonance frequency of C3 with different adjacent groove spacing (*n* = 3, $g_{2\;}$ = 3.5 μm, $t_{i}\;$ = 3.5 μm ).

For the slotted membrane, the radial position of the grooves mainly depended on the width, number of grooves, and spacing of adjacent grooves, which were chosen using the single variable method. When the number of grooves was determined, the effective thickness and stiffness of the membrane were determined by the width of the grooves and the spacing of adjacent grooves. Hence, a wider groove and smaller spacing between adjacent grooves were preferred to reduce the average thickness and stiffness of the membrane, which enhances the deformation capacity of the membrane. Assuming that the number of grooves was 3 and the spacing of adjacent grooves was 2 μm, C3 with grooves with a width of 2–7 μm was simulated under pressures of 0–10 kPa. As shown in Figure 9a, the pressure sensitivity improved as the groove width increased, but the growth rate began to slow down at 3.5 μm. Subsequently, assuming that the number of the grooves was 3 and the groove width was 3.5 μm, the results in Figure 9b reveal that the pressure sensitivity reached a maximum when the adjacent groove spacing was 2 μm. In summary, the groove width and adjacent groove spacing were 3.5 μm and 2 μm, respectively.

**Figure 10:**
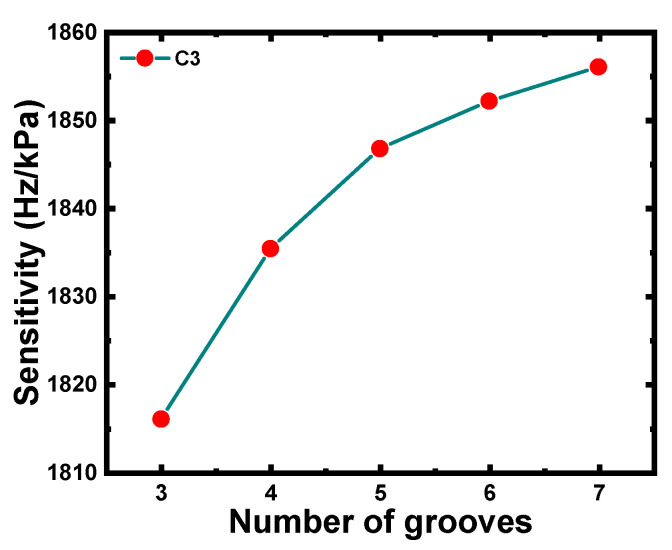
Pressure sensitivity of C3 with different numbers of grooves ($t_{i\;}$= 3.5 μm, $g_{1\;}$ = 3.5 μm, $g_{2}\;$ = 3.5 μm ).

Finally, C3 with groove numbers of 3, 4, 5, 6, and 7 were simulated to characterize the vibration tendency of the CMUT. The curve in Figure 10 demonstrates that the pressure sensitivity increased as the number of grooves increased; however, the curve gradually tended to be smooth, which indicated that the closer the grooves were to the center of the membrane, the slower the growth rate of the pressure sensitivity. Due to the complexity of the actual manufacturing process, a 5-groove design was preferred.

Based on the above, slotting diversity, that is, the non-uniformity of groove widths and different distribution of grooves, was investigated. For a constant total groove width, the distributions of the groove widths were $s_{1}\;$= 5.5 μm, $s_{2\;}$= 4.5 $\mu m,s_{3}\;$= 3.5 $\mu m,s_{4}\;$= 2.5 $\mu m,s_{5}\;$= 1.5 μm and $u_{1}\;$= 1.5 $\mu m,u_{2}\;$= 2.5 $\mu m,u_{3}\;$= 3.5 $\mu m,u_{4\;}$= 4.5 $\mu m,u_{5\;}$= 5.5 μm for C4 and C5, respectively. The corresponding results are shown in Section 3.2.

In theory, the collapse voltage is inversely proportional to the stiffness of the membrane, and after introducing slots, the collapse voltage of C3–C5 would decrease. The data in Table 3 verifies that compared with C1, the collapse voltages of C3–C5 were dramatically reduced, and the difference in slotting modes had little impact on the collapse voltage.

### 3.2. Comparison and Discussion

According to Section 3.1, the optimized geometrical parameters of C1–C5 are listed in Table 4 and Table 5. The data in Table 4 are the public parameters of C1–C5, and the data in Table 5 are the appropriative parameters for C2–C5. In this section, the displacement, resonant frequency, pressure sensitivity, and linear fit of C1–C5 are compared to adequately demonstrate the superiorities of the new designs.

**Figure 11:**
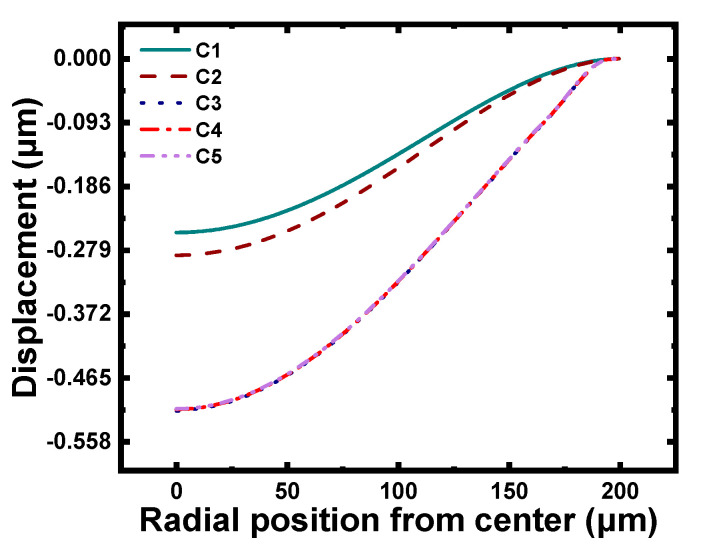
Displacement curves of C1–C5.

First, the curves in Figure 11 show that compared to C1, the displacement of C2–C5 increased. However, there was a significant discrepancy between C2 and C3–C5. The information in Table 6 shows that the maximum displacement of C2 and C3–C5 were approximately 0.29 μm and 0.51 μm, respectively, and increased by approximately 16% and 104%, respectively.

**Figure 12:**
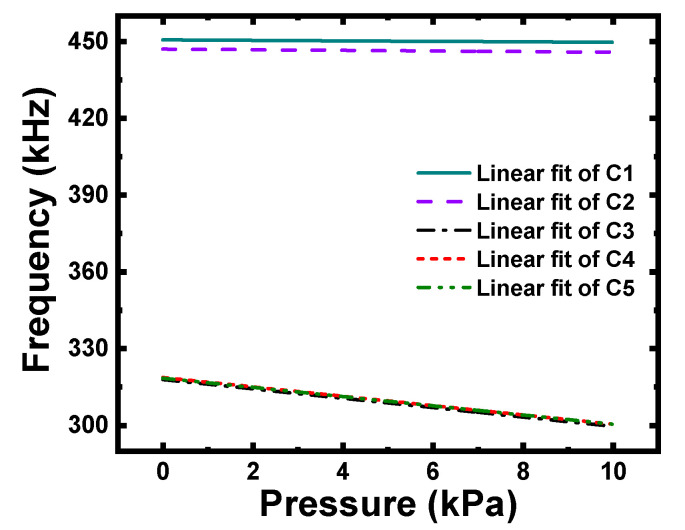
Resonance frequency of C1–C5 under pressures of 0–10 kPa.

Second, as shown in Figure 12, the resonance frequencies of C2 and C3–C5 decreased (with C3–C5 being more noticeable), which meant that the round-table cavity and the slotted membrane would partly hinder the realization of a higher-frequency CMUT.

Third, the data in Table 7 shows that the pressure sensitivities of C2, C3, C4, and C5 were 122.17 Hz/kPa, 1832.06 Hz/kPa, 1828.50 Hz/kPa, and 1797.61 Hz/kPa, respectively, and increased by 30.79%, 1861.31%, 1857.50%, and 1824.43%, respectively; these findings reveal that the frustum cone-like cavity and the slotted membrane immensely improved the pressure sensitivity of the CMUT. Furthermore, the data in Table 7 also shows that the linear fit of C2–C5 increased by 0.17%, 0.30%, 0.30%, and 0.30%, respectively, indicating that the two designs had some correction function on the nonlinearity of the micro-pressure measurement.

Finally, it is clear that the displacement curves of C3, C4, and C5 almost coincided, as shown in Figure 11, and the data in Table 5 also indicates that the maximum displacements of C3–C5 were identical. In addition, the linear fit lines of C3, C4, and C5 in Figure 12 effectively coincide, and the pressure sensitivity and linear fit in Table 7 show little difference. These results fully demonstrate that the uniformity of grooves and the distribution of grooves were unrelated to the performance of the CMUT; this finding will greatly enrich the design flexibility of the membrane structure.

In order to confirm the rationality of the results above, this section compares them with the experimental results of past studies, such as for displacement, collapse voltage, sensitivity, etc., as shown in Table 8. It is worth noting that these studies effectively reduced the stiffness of the membrane, through the method of grooving the membrane, and achieved an increase in the displacement of the membrane, which caused a reduction of the collapse voltage and an improvement of the measurement sensitivity. Among them, the structure of the pressure sensor made in [21] is the same as that of the traditional CMUT in this paper. Therefore, the sensitivities of the two are similar, and the difference of parameters is mainly caused by the parameters of the membrane. The experimental results of the condenser microphones prepared in [32,33] differ greatly. Compared with the results measured in [32], the displacement, collapse voltage, and sensitivity in [33] are 19.6, 0.2, and 15.8 times higher, respectively. These changes are mainly due to the C-shaped grooves on the edge of the wafer perforated diaphragm. For the MEMS condenser microphone prepared in [34], the displacement, collapse voltage, and sensitivity of the diaphragm after introducing slots were 2.7, 0.6, and 7.3 times higher than those beforehand. These findings demonstrate the same trend of change as in the present paper, wherein the displacement, collapse voltage, and sensitivity were 2, 0.7, and 19.6 times higher, respectively. The difference in the increase range is mainly caused by the difference of the grooves.

## 4. Conclusions

This paper presents two novel CMUTs for micro-pressure detection in the range of 0–10 kPa by modifying the cavity to a frustum cone-like cavity and adjusting the membrane to a slotted membrane. The working principle was to utilize the linear relationship between the resonance frequency and pressure applied to the membrane. Supported by theoretical analysis and FEA, the geometric parameters and characteristics of the traditional CMUT, CMUT with the frustum cone-like cavity, and CMUT with the slotted membrane were optimized. The results are as follows:

The maximum displacement, resonance frequency, pressure sensitivity, and R-square of the traditional CMUT were 0.25 μm, 450.63 kHz, 93.41 Hz/kPa, and 0.9968, respectively; these figures serve as a point of comparison for the experimental CMUTs. For the frustum cone-like cavity, the results clearly showed that the maximum displacement, pressure sensitivity, and R-square were 0.2912 μm, 122.17 Hz/kPa, and 0.9985, respectively, demonstrating increases of 16.01%, 30.79%, and 0.17%, respectively. For the slotted membrane, the maximum displacement, pressure sensitivity, and R-square were 0.5126 μm, 1832.06 Hz/kPa, and 0.9998, respectively, demonstrating increases of up to 104.22%, 1861.31%, and 0.30%, respectively. These data reveal that the two modified structures could promote the deformability and the measurement performance of the CMUT. In addition, the uniformity of the width of the grooves was determined to be unrelated to the characteristics of the membrane. In summary, this research establishes a solid foundation and provides a new direction of development for the precise measurement of micro-pressure.

## Figures and Tables

**Table 1:** Properties of materials in the finite element model (FEM).

	$\rho /kg\;m^{-3}$	E/GPa	v	$\varepsilon_{\gamma }$
Top electrode (Au)	19,300	70	0.44	-
Membrane (Si)	2332	169	0.29	11.7
Vacuum gap	-	-	-	1
Insulation (SiO_2_)	2200	70	0.17	4.2

**Table 2:** Pressure sensitivity and linear fit of C3 with a single 2 μm width groove.

Depth of Groove/μm	Sensitivity/(Hz/kPa)	R-Square
0	93.42	0.9963
0.5	107.84	0.9970
1	137.89	0.9980
1.5	196.02	0.9988
2	299.82	0.9994
2.5	477.34	0.9995
3	751.41	0.9997
3.5	1006.40	0.9999
4	1208.23	0.9999
4.5	1132.88	0.9999

**Table 3:** The collapse voltages of C3–C5.

	C3	C4	C5
$V_{collapse\;}$(V)	298	299	299

**Table 4:** Common structural parameters of C1–C5.

	$\mathrm{Radius}\;r_{m}\;\left(\mu m\right)$	Thickness h (μm)	$\mathrm{Cavity}\;\mathrm{Height}\;d_{0}\;(\mu m)$
C1–C5	200	5	3

**Table 5:** Different structural parameters of C2–C5 (units: μm ).

C2	C3–C5		C3	**C4**					**C5**				
$r_{c}$	$g_{1}$	$g_{2}$	$t_{i}$	$s_{1}$	$s_{2}$	$s_{3}$	$s_{4}$	$s_{5}$	$u_{1}$	$u_{2}$	$u_{3}$	$u_{4}$	$u_{5}$
50	2	3.5	3.5	5.5	4.5	3.5	2.5	1.5	1.5	2.5	3.5	4.5	5.5

**Table 6:** The maximum displacement and enhancement of C1–C5.

	The Maximum Displacement/μm	Enhancement
C1	0.2510	-
C2	0.2912	16.01%
C3	0.5126	104.22%
C4	0.5128	104.30%
C5	0.5132	104.46%

**Table 7:** Pressure sensitivity, linear fit, and enhancement of C1–C5.

	Sensitivity (Hz/kPa)	Enhancement	R-Square	Enhancement
C1	93.41	-	0.9968	-
C2	122.17	30.79%	0.9985	0.17%
C3	1832.06	1861.31%	0.9998	0.30%
C4	1828.50	1857.50%	0.9998	0.30%
C5	1797.61	1824.43%	0.9998	0.30%

**Table 8:** Comparison between this work and other works.

Ref.	Structure of Membrane	Size of Membrane	Thickness of Membrane	Frequency	Displacement	Collapse Voltage	Sensitivity
[21]	Not slotted	400 μm	5 μm	200 kHz	0.53 μm	>500 V	50.57 Hz/kPa
[32]	Not slotted	0.5 mm	3 μm	-	0.34 nm	63 V	0.2 m V/Pa
[33]	Slotted	0.38 mm	5 μm	70 kHz	6.67 nm	10.47 V	3.16 m V/Pa
[34]	Not slotted	2.43 mm	3 μm	1.11 MHz	0.245 μm	214 V	5.33×10^−6^ pF/Pa
	Slotted	1.5 mm	3 μm	528.57 kHz	0.6643 μm	120 V	3.87×10^−5^ pF/Pa
This work	Not slotted	200 μm	5 μm	450.63 kHz	0.25 μm	454 V	93.41 Hz/kPa
	Slotted	200 μm	5 μm	299.08 kHz	0.51 μm	298.4 V	1832.06 Hz/kPa
